# Comparative Evaluation of the Antimicrobial Efficacy of Chemical and Phytomedicinal Agents When Used As Intracanal Irrigants: An In Vitro Study

**DOI:** 10.7759/cureus.48754

**Published:** 2023-11-13

**Authors:** Ravinder K Gulati, Neha Jain, Anjali Singh, Anushree Jain, Avnish Jindal, Mahendra K Kumawat, Kapil Paiwal

**Affiliations:** 1 Department of Pedodontics and Preventive Dentistry, Daswani Dental College & Research Center, Kota, IND; 2 Department of Prosthodontics, Daswani Dental College & Research Center, Kota, IND; 3 Department of Conservative Dentistry and Endodontics, Daswani Dental College & Research Center, Kota, IND; 4 Department of Oral and Maxillofacial Surgery, Daswani Dental College & Research Center, Kota, IND; 5 Department of Oral and Maxillofacial Pathology, Daswani Dental College & Research Center, Kota, IND

**Keywords:** irrigation, metronidazole, babool, propolis, 2% chlorhexidine gluconate

## Abstract

Aim: This study aimed to compare and evaluate the antimicrobial efficacy of chemical and phytomedicinal agents when used as intracanal irrigants against *Candida albican (C. albican) and Enterococcus faecalis (E. faecalis).*

Materials and methods: This study was conducted at Kothiwal Dental College and Research Centre, Moradabad, India. One-hundred human tooth roots with a standardized length of 12±0.5 mm were divided into two groups (A and B, 50 each) inoculated with *C. albican *and *E. faecalis, *respectively. The groups were further divided according to the irrigants: A1 (11% ethanolic extract of propolis), A2 (2% chlorhexidine gluconate (CHX)), A3 (0.5% metronidazole), A4 (10% babool), and A5 (sterile saline (control) for the *C. albican *group and *E. faecalis* group (B1 to B5, respectively). The samples of different specimens were taken at subsequent intervals. The first collection was taken two days and 21 days after inoculation in group A and group B, respectively. The second collection was taken post irrigation, and the third^ ^collection seven days after different irrigants were used in both the groups. Microbiological samples were grown in a culture medium and incubated at 37°C for 24 hours for *C. albican* and 48 hours for *E. faecalis *(Sabouraud dextrose agar for *C. albican* and brain heart fusion for *E. faecalis*). The results were submitted for analysis of variance (ANOVA) and post-hoc test for statistical analysis.

Results: In group A, 2% chlorhexidine gluconate showed a highly significant percentage reduction of colony-forming unit (CFU) count (p≤0.001) with respect to the time interval against *C. albican,* followed by metronidazole, babool, propolis, and saline, whereas in group B, propolis showed a significant percentage reduction of CFU count (p<0.001) with respect to time interval against *E. faecalis, *followed by 2% CHX, metronidazole, babool, and saline.

Conclusion: Two percent chlorhexidine gluconate showed the highest antimicrobial efficacy against *C. albican,* whereas propolis showed the highest antimicrobial efficacy against *E. faecalis*. Chemical irrigants proved effective over herbal irrigants against *C. albican,* whereas herbal irrigants showed better antimicrobial efficacy over chemical irrigants against *E. faecalis*.

## Introduction

Metabolic products of microorganism play a keen role in causing pulp and periapical lesion, which eventually turns into pulp necrosis and inflammatory reactions [1]. The root canal infections are polymicrobial in nature. The most important of which is *Enterococcus faecalis* [2]. In addition to these microorganisms, some yeast-like microorganisms have also been found to be associated with secondary endodontic infections, particularly Candida albican [3]. Because of its physiochemical and biofilm forming property E. faecalis have been able to survive in surrounding environmental and nutritional conditions [4]. Most commonly it is found in endodontically treated cases (22-77%) [5]. C. albican are the most commonly found fungi, ranging from 7-18% of the infections [6]. The hyphal formation and thigmotropism (turning and bending of microorganism in response to touch stimulus) property allow C. albican to penetrate deep into the dentinal tubules, and also phenotypic alteration of C. albican helps to adapt in ecologically harsh conditions as in high alkaline environment [7].

Bacterial neutralization and toxin inactivation with a proper method of instrumentation and irrigating solutions is a key to the successful treatment. Chlorhexidine gluconate (CHX), a bis-biguanide (a compound that has a bactericidal property), has routinely been used as an effective antibacterial solution in dentistry, but it lacks tissue-dissolving activity in endodontics. CHX is used in two concentrations (0.2% and 2%) [8]. Metronidazole is a main antibiotic used in the treatment of anaerobic infections. Its mechanism of action is not entirely clear, but it usually interferes with the DNA synthesis. It is widely used in root canal disinfection and has been found successful in making the canal sterile [9]. Due to the increased microbial resistance and harmful and toxic effect of these chemical antibacterial agents, there is a need for herbal agents that should be affordable, less toxic, and effective [10]. Propolis is one such herbal product that has both antibacterial and antifungal properties. It is resinous substance yellow to dark brown in color used by bees to seal their unwanted hive spaces from outside contamination [11]. Higher temperature, small size, and wetness of the hive make it an ideal place for bacterial growth; however, microorganisms do not grow because of the antibacterial properties of propolis. The antibacterial property is attributed to flavonoids and aromatic compounds, such as caffeic acid [12].

Babool has got higher concentration of methionine, cysteine, threonine, lysine, tryptophan, potassium, phosphorus, magnesium, iron, and manganese. It possesses good antimicrobial activity, antioxidant activity antifungal, and antiviral activity. The antimicrobial potency of plants is believed to be due to tannins, saponins, phenolic compounds, essential oils, and flavonoids. Because of so many effective ingredients found in herbal agents, it was concluded that these agents could be used as effective endodontic irrigants [13]. According to the literature, few studies have performed a comparison of the antimicrobial efficacy of the above-mentioned herbal agents with chlorhexidine gluconate and metronidazole as intracanal irrigants.

Thus, this in-vitro study was conducted with an aim to compare and evaluate the antimicrobial efficacy of propolis and babool with chlorohexidine gluconate and metronidazole taking saline as a control when used as intracanal irrigants against *C. albican* and *E. faecalis*.

## Materials and methods

This study was conducted at Kothiwal Dental College and Research Centre, Moradabad, India. Ethical approval was obtained from its Institutional Ethical Review Board (IERB) with approval number IERB/06/2017/05.

Tooth sample preparation 

Small sample sizes of 100 single-rooted human teeth extracted for periodontal disease were cleaned and washed in saline before use. The inclusion/exclusion criteria notified below were considered during the selection process. Single-rooted extracted teeth, no pathological resorption of the roots, and caries-free roots were selected for the study, whereas multirooted teeth and pathologically resorbed and carious roots were excluded from the study. Removing the crown portion, the remaining length of the root was standardized at 12±0.5 mm. The instrumentation was done 0.5 mm beyond the apex by a mean of a #25K file followed by instrumentation to 1 mm short of apex with a #30 K-file. Ethylenediaminetetraacetic acid (EDTA) (17%) was then used to fill the root canals for three minutes, followed by rinsing with 5 ml of saline solution. The apex was closed using 3M ESPE Filtek Z350 XT composite resin (3M Company, USA), and the roots were embedded in a self-cure acrylic except for the root canal opening. The selected specimens were divided into two groups (group A and group B), with 50 teeth in each group. In group A, antimicrobial efficacy of used irrigants was tested against *C. albican*. In group B, antimicrobial efficacy of irrigants was tested against *E. faecalis*.

Preparation of species

**Figure 1:**
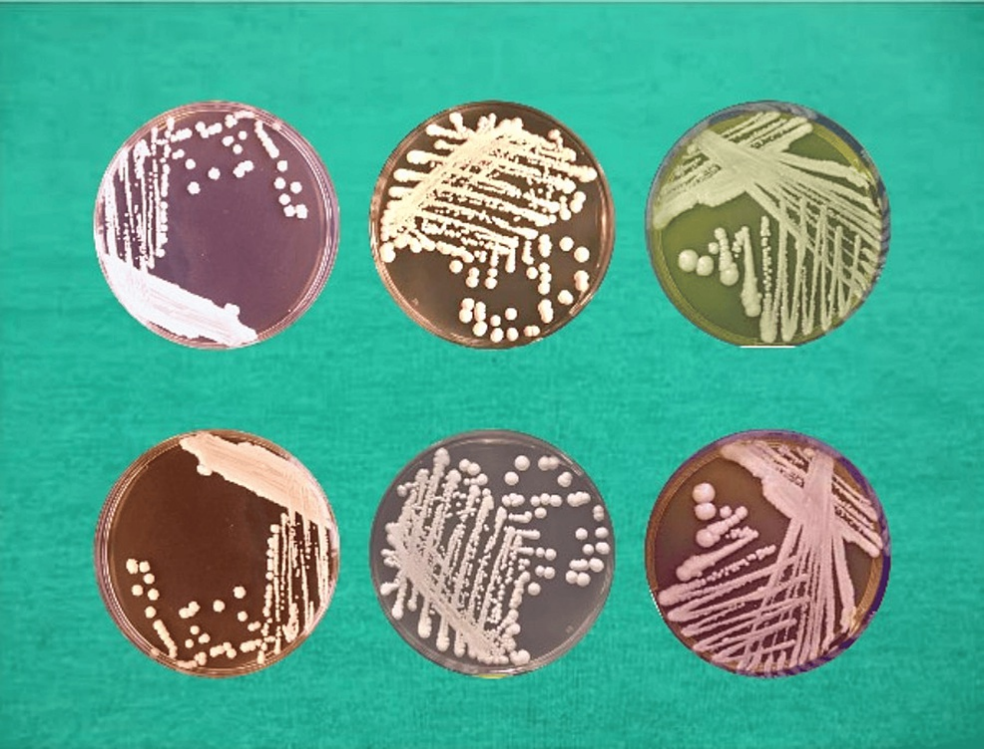
Confirmatory collection of Candida Albican (after inoculation)

**Figure 2:**
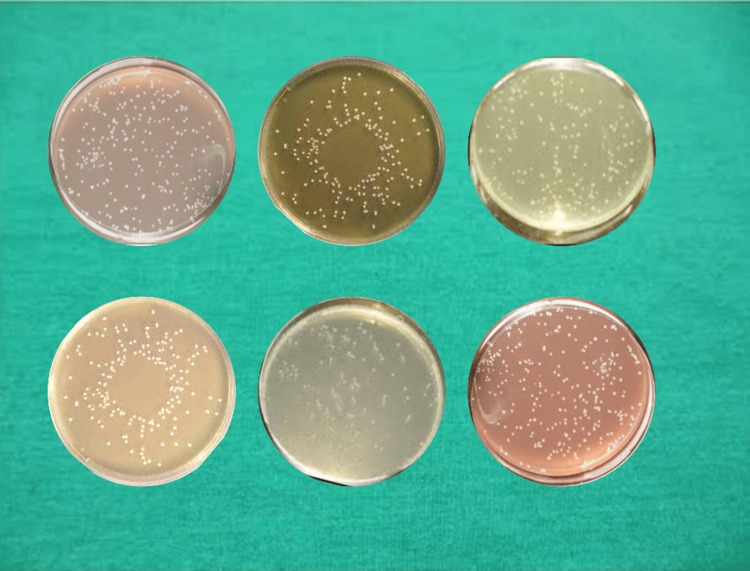
Confirmatory collection of Enterococcus faecalis (after inoculation)

The microorganism’s strains used were *C. albican* (MTCC 227) and *E. faecalis* (MTCC 439). Both microorganisms were seeded on Petri dishes containing Sabouraud dextrose agar (SDA) for *C. albican* and brain heart infusion (BHI for *E. faecalis*). The SDA dishes were incubated in a bacteriological oven at 37±1°C for 24 hours, while the BHI dishes were incubated for a period of 48 hours. Standardized saline solution suspensions of *C. albican* and *E. faecalis* were prepared (108 cells/mL). The root canals of group A were inoculated with 10 μL of *C. albican* and Sabouraud dextrose broth each, and group B root canals were inoculated with 10 μL of *E. faecalis* and BHI, each resulting in 20 ul of inoculated medium in each root canal in the respective groups. A sterile cotton pellet embedded in the respective broth was placed at the entrance of the canals. Group A samples were stored in an incubator at 37±1ºC in a humid medium for two days and group B samples for 21 days. A small amount of BHI broth was poured after every three days for 21 days. Once the incubation period for each group was completed, the samples of all the specimens were collected and counted using a digital colony counter (Obst1) (Figures 1, 2).

The confirmatory collection of *E. faecalis* (after inoculation) is shown in Figure 2.

The specimens in each group were further divided into five subgroups according to the irrigants used: group A (*C. albican*, A1: 11% propolis, A2: 2% chlorhexidine gluconate, A3: 0.5% metronidazole, A4: 10% babool, A5: saline) and group B (*E. faecalis*, B1: 11% propolis, B2: 2% chlorhexidine gluconate, B3: 0.5% metronidazole, B4: babool, B5: saline).

Canals of all the specimens of each subgroup were sequentially prepared using the step-back technique up to size #30 master apical file, flaring the canal was performed up to size #50, and 3 ml of the experimental intracanal irrigants in each subgroup were used after each file used for instrumentation. Microbiological samples were then collected immediately post-irrigation (second collection) and seven days post-irrigation (third collection). The collection method of microbial sampling was as follows.

Use of a sterile paper point was done for collecting microbial samples. No. 30 paper point was standardized, which was placed in the root canals for almost one minute and further dipped in a test tube filled with 5 ml of a sterile saline solution. With this, the paper point was stirred for about 30 seconds. About 0.1 mL of the solution prepared was poured in two separate dishes containing SDA for *C. albican* and BHI for *E. faecalis*. The second microbial collection was done after seven days, but till then, the root canals of all the specimens were filled with a sterile saline solution, and the root canal opening was closed with the help of a sterile cotton pellet. All the samples were then placed in the incubator at 37°C.

The culture plates were incubated at 37°C for 48 hours, and the number of colonies was then counted using a colony counter for *C. albican* and *E. faecalis*. The colonies of *C. albican* were identified based on their colony character, which appeared microscopically as cream-colored raised cocci-forming striations and *E. faecalis* as spheres or cocci, and the obtained data were subjected to statistical analysis.

## Results

One-way analyses of variance (ANOVA) and post-hoc test were used to assess the statistical significance between groups and within the group. In the above test, a p-value less than 0.05 was taken to be statistically significant. The results were assessed on the percentage reduction of colony-forming units (CFUs) with respect to the time interval. The data were analyzed using IBM SPSS Statistics version 16 for Windows 10.5 (released 2007, IBM Corp., Armonk, New York, United States).

The distribution of mean CFUs per mL (CFU/mL) for each subgroup against *C. albican* and *E. faecalis* were determined, as shown in Table 1 and Table 3 and Table 2 and Table 3, respectively. The percentage reduction of CFUs per mL with respect to time interval against *C. albican* and *E. faecalis* were statistically determined, as shown in Table 2 and Table 4. There was a statistically highly significant (p≤0.001) difference among all the subgroups against *C. albican* and *E. faecalis* (Table 1).

**Table 1:** Distribution of mean±standard deviation of the colony-forming unit (CFU) count of Candida albican with respect to time interval in subgroups

Group A	Means and standard deviation		
Subgroups (n=10)	ObsT1	ObsT2	ObsT3
Propolis	7.48±0.717	6.659±0.673	5.175±0.944
2% chlorohexidine	7.667±0.505	4.031±0.860	0.811±0.377
0.5% metronidazole	7.462±0.72	5.406±0.82	2.902±1.36
10% babool	7.388±0.653	5.93±0.445	3.91±0.833
Saline	7.808±0.668	7.673±0.657	7.13±0.732

The percentage reduction of the CFU count of *C. albican* with respect to time interval in subgroups is presented in Table 2.

**Table 2:** Percentage reduction of the CFU count of Candida albican with respect to time interval in subgroups

Group A	Subgroup	%reduction1	p-value	%reduction2	P-value
Candida albican	Propolis	10.67	<0.001	29.84	<0.001
	2% chlorohexidine	47.62		89.60	
	Metronidazole	26.49		59.96	
	10% babool	19.45		47.10	
	Saline	1.73		8.68	
	Total	21.19		47.03	

The distribution of mean±standard deviation of the CFU count of *E. faecalis* with respect to time interval in subgroups is presented in Table 3.

**Table 3:** Distribution of mean±standard deviation of the CFU count of E. faecalis with respect to time interval in subgroups

Group B	Means and standard deviation		
Subgroups (n=10)	ObsT1	ObsT2	ObsT3
Propolis	6.01±0.517	4.886±0.612	3.114±0.964
2% chlorohexidine	6.112±0.54	5.254±0.658	4.133±0.698
0.5% metronidazole	6.387±0.472	5.859±0.438	5.401±0.472
10% babool	6.077±0.506	5.807±0.561	5.459±0.491
Saline	6.273±0.328	6.059±0.293	5.943±0.295

The percentage reduction of the CFU count of *E. faecalis* with respect to time in subgroups is presented in Table 4.

**Table 4:** Percentage reduction of the CFU count of E. faecalis with respect to time in subgroups

Group B	Subgroup	%reduction1	p-value	%reduction2	p-value
Enterococcus faecalis	Propolis	18.69	<0.001	48.31	<0.001
	2% chlorhexidine gluconate	14.19		32.58	
	Metronidazole	8.13		15.49	
	10% babool	4.51		10.18	
	Saline	3.39		5.24	
	Total	9.78		22.36	

**Figure 3:**
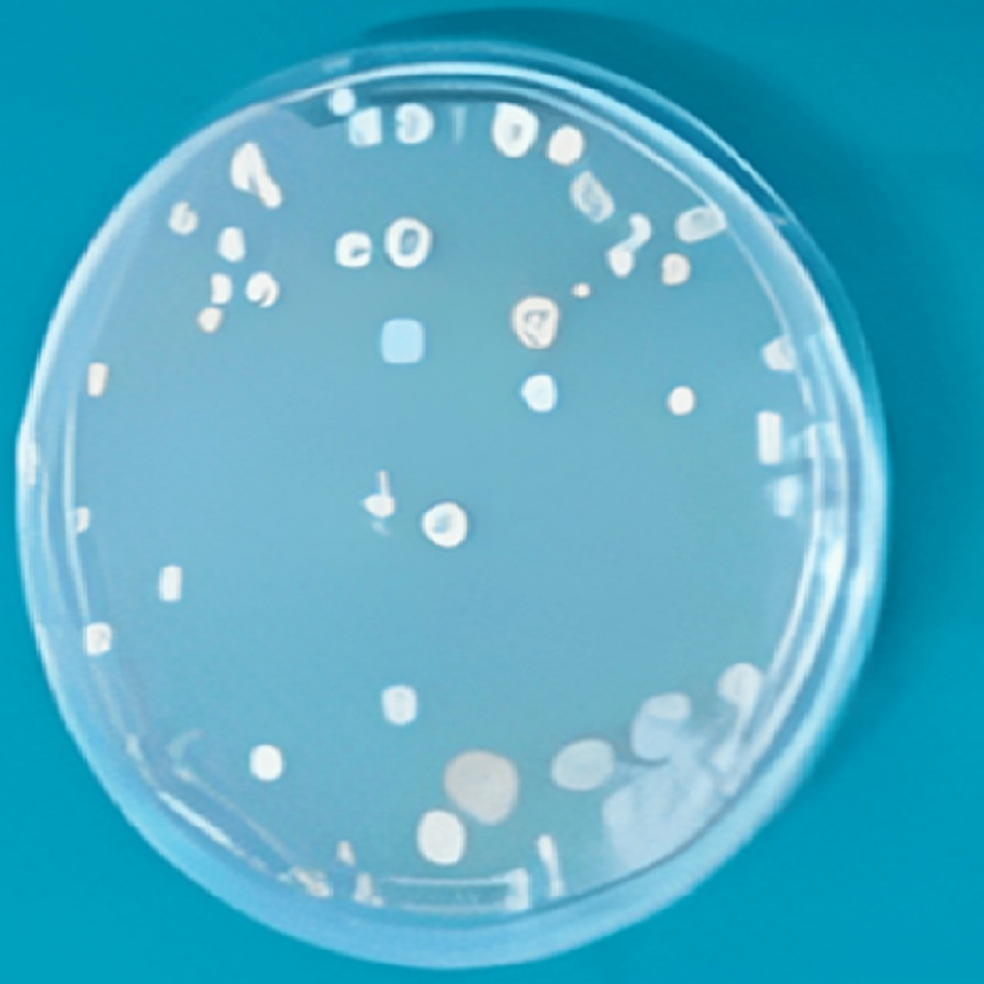
Maximum reduction in CFU count in CHX group against candida albican after 7 days of irrigation

The mean% reduction of CFUs with respect to time interval for CHX against *C. albican* was 47.62% (ObsT1-ObsT2) and 89.60% (ObsT1-ObsT3). Against *E. faecalis*, the mean% reduction of CFUs with respect to time interval was 14.19% (ObsT1-ObsT2) and 32.58% (ObsT1-ObsT3). CHX had shown maximum antimicrobial efficacy against* C. albican* among all the subgroups used, whereas against *E. faecalis*, the CFU count reduction was less compared to propolis (ObsT1-ObsT2, 18.69% and Obst1-ObsT3, 48.31%), showing a statistically highly significant difference (p<0.001) (Figure 3).

**Figure 4:**
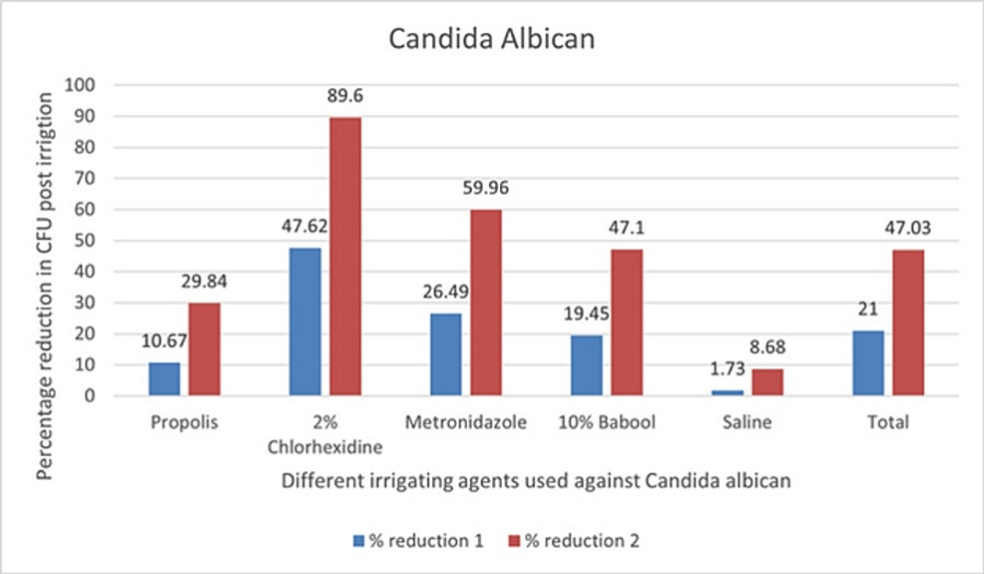
Graph of percentage reduction of CFU count of Candida albican with respect to time interval in individual groups

The graph of percentage reduction of the CFU count of *C. albican* with respect to time interval in individual groups is presented in Figure 4.

**Figure 5:**
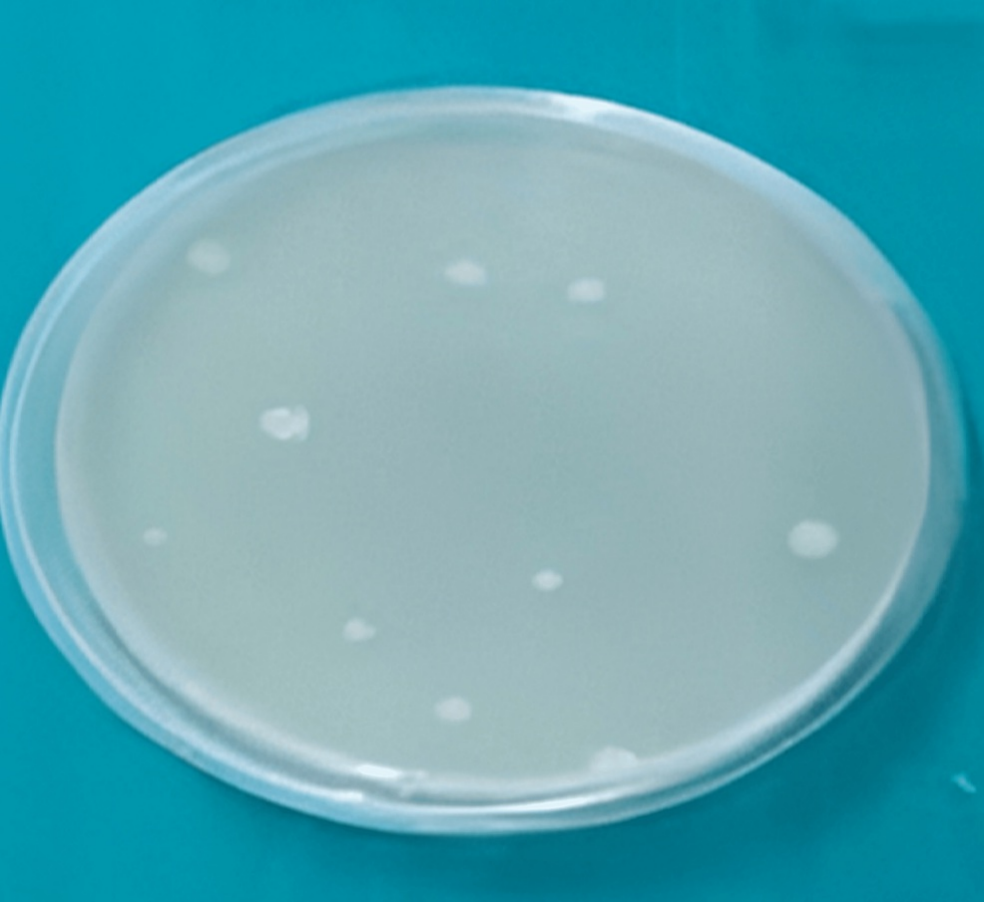
Maximum reduction in CFU count in propolis group against E. faecalis after 7 days of irrigation

The mean% reduction of CFUs with respect to time interval for propolis against *C. albican* (ObsT1-ObsT2) was 10.67% and that for ObsT1-ObsT3 was 29.84%. The mean% reduction of CFU with respect to time interval against *E. faecalis* (ObsT1-ObsT2) was 18.69% and that for ObsT1-ObsT3 was 48.31%, respectively. According to the results, the CFU count was reduced immediately after the irrigation and seven days after irrigation with propolis. Among all the subgroups used against *E. faecalis*, propolis has shown the maximum antimicrobial efficacy compared with other groups (Figure 5), whereas against *C. albican,* it had significantly reduced the CFU count but less as compared to babool and the chemical irrigants used.

**Figure 6:**
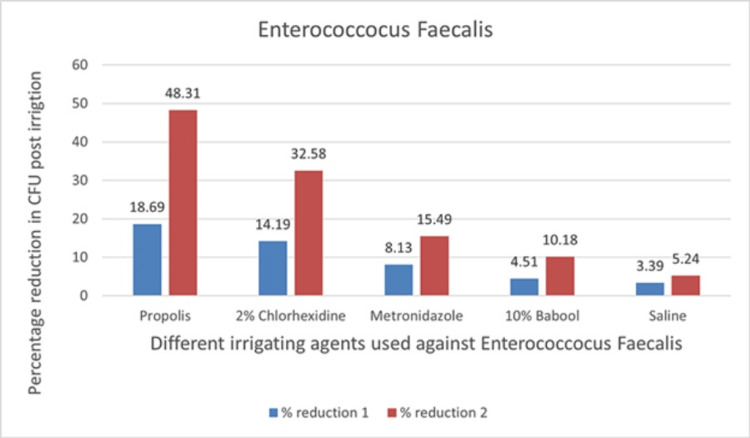
Graph of percentage reduction of CFU count of E. faecalis with respect to time interval in all subgroups

The graph of percentage reduction of the CFU count of *E. faecalis* with respect to time interval in all subgroups is shown in Figure 6.

## Discussion

*Enterococcus* species habitat in large quantities in the intestinal lumen of human beings, and under normal conditions, they cause no harm to their host [14]. *E. faecalis* has been frequently isolated from infected pulps and persistent infections in post-endodontic treatments [15]. They oxidize many energy sources, such as carbohydrates, glycerol, lactate, malate, citrate, arginine, and many keto acids. Enterococci can make themselves adapt to harsh environmental conditions, including very extreme alkaline pH (9.6) and salt concentration, have the capacity to withstand a high-degree temperature of 60 °Cfor 30 minutes, and also grow in the temperature range of 10-45°C. Enterococci can also resist bile salts, detergents, heavy metals, ethanol, azide, and desiccation [16]. In addition, yeast-like microorganisms have been found to be associated with secondary endodontic infections, particularly *C. albican*. *C. albican* releases collagenolytic enzymes that make it possible to use dentin as a nutrient source. Waltimo et al. [9] reported the presence of *C. albican* in pure cultures in therapy-resistant apical periodontitis, establishing it as a plausible cause of root canal failure.

In this study, the dentin block model method was used, which is considered the most standard method, and a statistical comparison is feasible [16,17]. Penetration into dentinal tubules is the most important resistance mechanism of *E. faecalis* and *C. albican* against antibacterial agents in endodontic treatments. This model provides reconstruction of the microanatomy of dentin, especially dentinal tubules. The dentin block model also simulates the chemical environment of the root canal and the ability of biofilm development [18]; therefore, this method was accepted as the method of choice. Using this method, the experimental species were inoculated, and the specimens were incubated for two days for *C. albican* and 21 days for *E. faecalis* [19]. Chlorhexidine gluconate over the period of time has shown great results during the biomechanical preparation because of its great antimicrobial efficacy against the microorganisms found in root canals [20]. It is because CHX gluconate is water soluble, and at physiologic pH, it readily dissociates and releases the positively charged CHX component. The interaction of the positive-charge and negative-charge phosphate groups on microbial cell walls alters the cell’s osmotic equilibrium [21]. This increases the permeability of the cell wall, which allows the CHX molecule to penetrate into the bacteria. The antifungal action is explained by the fact that it leads to the coagulation of nucleoproteins and changes in the cell wall, allowing the possible escape of cytoplasmic components through plasmalemma, and is also capable of inhibiting the *C. albican* adhesion to biological and inert surfaces [22].

Oncag et al. [23] found that after five minutes and 48 hours, 2% CHX showed reduction in CFU count against *E. faecalis*. The results of our study are in accordance with their study, where the CFU count also reduced immediately after irrigation with CHX and seven days after irrigation.

Among the chemical irrigants used, metronidazole also proved to be effective against *C. albican* over herbal irrigants but less compared to 2% CHX. Metronidazole is bactericidal against most anaerobes that contain electron transport components, such as ferredoxin, which donate electrons to metronidazole, forming highly reactive nitro radical anions that kill susceptible organisms by a radical-mediated mechanism [24]. Topoisomerase II is homologous of bacterial gyrase found in fungus, which is the primary physiological target responsible for quinolone cytotoxicity and kills the cells by converting the type II enzyme into cellular poison [25]. Metronidazole had also effectively reduced the *E. faecalis* count immediately after irrigation and seven days after irrigation, but propolis and CHX had shown better antimicrobial efficacy against *E. faecalis*. Shweta et al. [26] stated that final flushing with 0.5% metronidazole after a hand or rotary instrument significantly reduced the CFU count, which was consistent with our study results.

The antimicrobial property of propolis is mostly attributed to flavonoids. In a study conducted by Mori et al. using radioactive precursors, they observed that flavonoids play a major role in the inhibition of DNA and RNA synthesis. It also has a role on protein and lipid synthesis inhibition but to a lesser extent. It is the B ring of the flavonoids that causes intercalation or hydrogen bonding that eventually leads to the stacking of nucleic acid bases and thus causes the inhibition action of DNA and RNA synthesis. The antifungal action of propolis studied by Mello et al. [27] is mainly because of its interaction with the cellular sulfhydryl compounds found in the cell wall of the microorganism, thereby disturbing its integrity. *Acacia nilotica* (L.), also known as Egyptian thorn, prickly acacia, gum Arabic, and babool, is a multipurpose nitrogen-fixing tree legume. It possesses good antimicrobial, antioxidant, anti-fungal, antiviral, antibiotic, anti-cancer, and anti-hypertensive activities. There are different varieties of babool used in leaves, flowers, stem, thorns, roots, gums, and fruits [28]. As stated above, there are different forms available, so in this study, we have used the fruit form of babool, which was first dried and then grinded.

In our study, 10% concentration of babool was used. It had shown a better antimicrobial efficacy against *C. albican* than propolis but less as compared to CHX. Comparable results were seen in the percentage reduction of CFU count with metronidazole, immediately after irrigation and also seven days after irrigation. Against *E. faecalis,* babool had also a reduced CFU count with respect to time interval but less as compared to propolis and CHX, whereas it had also shown comparable results with metronidazole. The antimicrobial potency of this plant is believed to be due to tannins, saponins, phenolic, essential oils, and flavonoids. Although babool has shown better antimicrobial efficacy against *C. albican* than *E. faecalis,* more studies are still required on babool against *E. faecalis* at different concentrations.

Normal saline was used as one of the experimental solutions, but it was taken as the control group in our study. Although saline had been used as a control group in our study, it also had shown significant reduction in microbial count post irrigation compared to the other irrigants used. This is consistent with the findings of Akpata [29] who stated that there was a reduction in the microbial count when saline was used as an irrigant, which is mainly attributed to its ability to flush debris from the root canal rather than having any antimicrobial property. Ohara et al. [30] showed that saline irrigants have been successful in significantly reducing the number of bacteria in the root canal but fail to completely eliminate it.

All the subgroups used against *C. albican* and *E. faecalis* had reduced the CFU count with respect to time interval. Although there were comparable results of metronidazole and babool against both the species, there was a statistically highly significant difference among all the subgroups (p<0.001). The results of this study acclaim the use of propolis and babool as root canal irrigants in permanent teeth, which might prove to be advantageous considering the several undesirable characteristics of CHX and metronidazole. Since the natural irrigants are less toxic when compared with chemical agents, plant-derived endodontic irrigants should be considered as promising agents to obtain root canals free of microbes and their by-products in acquiring a successful endodontic treatment.

Limitations

In the present study, two chemical and two herbal agents were compared against the experimental species. The study was conducted for 21 days. Although they have shown good antimicrobial efficacy against the species, better results could have been obtained if the study was done for a longer duration of time and the sample size taken was small for such a kind of studies. A larger sample size should be taken for more reliable and significant results. Moreover, while counting the CFU count using a digital colony counter, there might be a possibility of technical faults, which eventually must have not given accurate results.

## Conclusions

From the above stated results, 2% chlorohexidine gluconate showed the highest antimicrobial efficacy against *C. albican* among all the tested irrigants and 11% propolis showed the maximum mean percentage reduction in CFU count with respect to the time interval among all the tested irrigants against *E. faecalis*. Thus, chemical irrigants proved effective over herbal irrigants against *C. albican,* but propolis as a herbal irrigant showed better antimicrobial efficacy against *E. faecalis.* Therefore, it can be used as an effective root canal irrigant against *E. faecalis*.

## References

[1] Sakamoto M, Siqueira JF Jr, Rôças IN, Benno Y (2008). Molecular analysis of the root canal microbiota associated with endodontic treatment failures. Oral Microbiol Immunol.

[2] Sundqvist G (1994). Taxonomy, ecology, and pathogenicity of the root canal flora. Oral Surg Oral Med Oral Pathol.

[3] Nair PN, Sjogren U, Krey G, Kahnberg KE, Sundqvist G (1990). Intraradicular bacteria and fungi in root-filled, asymptomatic human teeth with therapy-resistant periapical lesions: a longterm light and electron microscopic follow-up study. J Endod.

[4] Distel JW, Hatton JF, Gillespie MJ (2002). Biofilm formation in medicated root canals. J Endod.

[5] Stuart CH, Schwartz SA, Beeson TJ, Owatz CB (2006). Enterococcus faecalis: its role in root canal treatment failure and current concepts in retreatment. J Endod.

[6] Waltimo TM, Haapasalo M, Zehnder M, Meyer J (2004). Clinical aspects related to endodontic yeast infections. Endod Topics.

[7] Zhang S, Wang QQ, Zhang CF, Soo I (2010). Identification of dominant pathogens in periapical lesions associated with persistent apical periodontitis. Chin J Dent Res.

[8] Kovac J, Kovac D, Slobodnikova L, Kotulova D (2013). Enterococcuss faecalis and Candida albicans in the dental root canal and periapical infections. Bratisl Lek Listy.

[9] Rossi S (2011). Australian medicines handbook.

[10] Ercan E, Zekinci T, Atakul F, Gul K (2004). Antibacterial activity of 2% chlorhexidine gluconate and 5.25% sodium hypochlorite in infected root canal: in vivo study. J Endod.

[11] Sonmez S, Kirilmaz L, Yucesoy M, Yücel B, Yilmaz B (2005). The effect of bee propolis on oral pathogens and human gingival fibroblasts. J Ethnopharmacol.

[12] Kandaswamy D, Venkateshbabu N, Gogulnath D, Kindo AJ (2010). Dentinal tubule disinfection with 2% chlorhexidine gel, propolis, morinda citrifolia juice, 2% povidone iodine, and calcium hydroxide. Int Endod J.

[13] Seigler DS (2003). Phytochemistry of Acacia-sensu lato. Biochem Syst Ecology.

[14] Gilmore MS (2002). The Enterococci: pathogenesis, molecular biology, and antibiotic resistance.

[15] Sen BH, Piskin B, Demirci T (1995). Observations of bacteria and fungi in infected root canals and dentinal tubules by SEM. Endod Dent Traumatol.

[16] Haapasalo M, Orstavik D (1987). In vitro infection and disinfection of dentinal tubules. J Dent Res.

[17] Siqueira JF Jr, de Uzeda M, Fonseca ME (1996). A scanning electron microscopic evaluation of in vitro dentinal tubules penetration by selected anaerobic bacteria. J Endod.

[18] Ercan E, Dalli M, Yavuz I, Ozekinci T (2006). Evaluation of microflora from failed roor canals and application of DMSO. Ann NY Acad Sci.

[19] Valera MC, Maekawa LE, de Oliveira LD, Jorge AO, Shygei É, Carvalho CA (2013). In vitro antimicrobial activity of auxiliary chemical substances and natural extracts on Candida albicans and Enterococcus faecalis in root canals. J Appl Oral Sci.

[20] Delany G, Patterson S, Miller C, Newton C (1982). The effect of chlorhexidine gluconate irrigation on the root canal flora of freshly extracted necrotic teeth. Oral Surg Oral Med Oral Pathol.

[21] Gomes BP, Vianna ME, Sena NT, Zaia AA, Ferraz CC, de Souza Filho FJ (2006). In vitro evaluation of the antimicrobial activity of calcium hydroxide combined with chlorhexidine gel used as intracanal medicament. Oral Surg Oral Med Oral Pathol Oral Radiol Endod.

[22] Ellepola ANB, Samaranayake LP (2001). Adjunctive use of chlorhexidine in oral candidosis: a review. Oral Dis.

[23] Onçağ O, Hoşgör M, Hilmioğlu S, Zekioğlu O, Eronat C, Burhanoğlu D (2003). Comparison of antibacterial and toxic effects of various root canal irrigants. Int Endod J.

[24] White RR, Hays GL, Janer LR (1997). Residual antimicrobial activity after canal irrigation with chlorhexidine. J Endod.

[25] Ozdek SC, Miller D, Flynn PM, Flynn HW (2006). In vitro antifungal activity of the fourth generation fluoroquinolones against Candida isolates from human ocular infections. Ocul Immunol Inflamm.

[26] Thakur S, Jain S (2013). Antibacterial efficacy of metronidazole and gluteraldehyde with hand and ultrasonic irrigation -an invitro study. UJDMS.

[27] Mello JCP, Petereit F, Nahrstedt A (1996). Prorobinetinidins from Sthryphnodendron adstringens. Phytochemistry.

[28] New TR (1984). A biology of acacias. A Biology of Acacias.

[29] Akpata ES (1976). Effects of endodontic procedures on the population of viable microorganisms in the infected root canal. J Endod.

[30] Ohara P, Torabinejad M, Kettering JD (1993). Antibacterial effects of various endodontic irrigants on selected anaerobic bacteria. Endod Dent Traumatol.

